# Liver magnetic resonance imaging, non-alcoholic fatty liver disease and metabolic syndrome risk in pre-pubertal Mexican boys

**DOI:** 10.1038/s41598-024-77307-8

**Published:** 2024-10-30

**Authors:** Benito de Celis Alonso, Elizabeth Shumbayawonda, Cayden Beyer, Silvia Hidalgo-Tobon, Briseida López-Martínez, Pilar Dies-Suarez, Miguel Klunder-Klunder, América Liliana Miranda-Lora, Eduardo Barragán Pérez, Helena Thomaides-Brears, Rajarshi Banerjee, E. Louise Thomas, Jimmy D. Bell, Po-Wah So

**Affiliations:** 1 Faculty of Physical and Mathematical Sciences, Benemérita Universidad Autónoma de Puebla, Puebla, Mexico; 2 Perspectum Ltd., Oxford, UK; 3 Imaging Department, Children’s Hospital of Mexico Federico Gómez, Mexico City, Mexico; 4 Physics Department, UAM Iztapalapa, Mexico City, Mexico; 5 Research Direction/Coordination, Hospital Juarez of Mexico, Mexico City, Mexico; 6 Epidemiological Research Unit in Endocrinology and Nutrition, Children’s Hospital of Mexico Federico Gomez, Mexico City, Mexico; 7 Neurology Department, Children’s Hospital of Mexico Federico Gomez, Mexico City, Mexico; 8 Research Centre for Optimal Health, University of Westminster, London, UK; 9 Department of Neuroimaging, King’s College London, London, UK

**Keywords:** Obesity, BMI, Metabolic syndrome, NAFLD, Multiparametric MRI, Young children, Non-alcoholic fatty liver disease, Metabolic syndrome, Dyslipidaemias, Obesity, Type 2 diabetes

## Abstract

Rising global pediatric obesity rates, increase non-alcoholic fatty liver disease (NAFLD) and metabolic syndrome (MetS) prevalence, with MetS being a NAFLD risk factor. NAFLD can be asymptomatic, with liver function tests insensitive to mild disease, and liver biopsy, risking complications. Thus, we investigated multiparametric MRI (mpMRI) metrics of liver fat (proton density fat fraction, PDFF) and disease activity (fibro-inflammation; iron-corrected T1, cT1), in a Hispanic pre-pubertal pediatric cohort, with increased risk of NAFLD. Pre-pubertal boys (n = 81) of varying Body-Mass Index (BMI) were recruited in Mexico City. Most children (81%) had normal liver transaminase levels, 38% had high BMI, and 14% had ≥ 3 MetS risk factors. Applying mpMRI thresholds, 12%, 7% and 4% of the cohort had NAFLD, NASH and high-risk NASH respectively. Participants with ≥ 3 MetS risk factors had higher cT1 (834 ms vs. 737 ms, p = 0.004) and PDFF (8.7% vs. 2.2%, p < 0.001) compared to those without risk factors. Those with elevated cT1 tended to have high BMI and high insulin (p = 0.005), HOMA-IR (p = 0.005) and leptin (p < 0.001). The significant association of increased risk of MetS with abnormal mpMRI, particularly cT1, proposes the potential of using mpMRI for routine pediatric NAFLD screening of high-risk (high BMI, high MetS risk score) populations.

## Introduction

Non-alcoholic fatty liver disease (NAFLD) is the leading cause of chronic liver disease worldwide^1,2^. NAFLD embodies a continuum of disease stages characterized by excessive lipid infiltration in hepatocytes (i.e., steatosis) that can advance via inflammatory and fibrotic pathways to more severe phenotypes such as non-alcoholic steatohepatitis (NASH) and liver cirrhosis^3^.

The global obesity rates have tripled in the past 50 years and are a major public health concern, with over 340 million children and adolescents being classified as overweight or obese; moreover, 5–10% of children with obesity also have NAFLD^1^. Although epidemiological studies typically indicate the rise in obesity to be highest in western developed countries such as the USA and UK^4^, industrialized developing countries such as Mexico are also showing a rapid increase in childhood obesity. The 2020 National Health Survey indicated that 19.6% of school children were overweight and 18.6% were obese in Mexico, with 26.8% of adolescents being overweight and 17% obese^5,6^. Crucially, obesity and NAFLD are also strongly associated with worse metabolic health and poor clinical outcomes, including increased likelihood of insulin resistance, Type 2 diabetes (T2DM), cardiovascular disease (CVD) and other chronic conditions^7^.

NAFLD is typically an asymptomatic disease and plasma biochemical liver health (function) markers are insensitive to mild disease^3,8^. It is of the utmost importance to identify individuals earlier along the disease continuum to prevent or slow progression to adverse clinical outcomes^8^. Liver biopsy, although recommended by clinical practice guidelines^9,10^ to support NAFLD/NASH diagnosis, is an imperfect reference standard with numerous well documented limitations^11–13^. Of particular concern in children, liver biopsy can result in pain, bleeding or infection^14^. Therefore, less invasive alternatives to liver biopsies are needed with similar diagnostic accuracy to support patient management and prognostication^15^. Currently, non-invasive technologies, such as ultrasound^16^ and elastography^17^, are being used to support patient management, however, these tests are predominantly validated only in adults. In addition, clinical guidance from the Expert Committee on NAFLD (ECON) and the North American Society of Pediatric Gastroenterology, Hepatology and Nutrition (NASPGHAN)^8^, as well as that from the British Society of Paediatric Gastroenterology, Hepatology and Nutrition (BSPGHAN)^18^, have highlighted the need for NAFLD identification in children. More specifically, clinical guidelines recommend earlier screening in younger children with MetS risk factors as they are at higher risk of long-term adverse clinical outcomes^8^. Non-invasive imaging assessments such as ultrasound and computerized tomography (CT) are clinically available. However, routine ultrasound has inadequate sensitivity and specificity to screen for NAFLD in children, and CT is not recommended in children due to radiation exposure^8^. Hence, NASPGHAN have suggested that MRI could be a viable testing modality for chronic pediatric liver disease.

Multiparametric magnetic resonance imaging (mpMRI) is increasingly being used to assess chronic pediatric liver disease. Proton density fat fraction (PDFF) can assess liver fat whereas iron-corrected T1 (cT1), a correlate of fibro-inflammation, can characterize disease activity^19^. Such mpMRI metrics have been able to identify sub-clinically active disease^19,20^ and support longitudinal patient monitoring^21^, including assessment of Fontan-associated liver disease^22^, and presence of radiologic portal hypertension in chronic progressive pediatric chronic liver diseases^23–25^. In adults with NAFLD/NASH, mpMRI has been able to predict adverse outcomes including all-cause mortality^26^, shown to be useful for monitoring treatment response^27–30^, aid patient understanding of chronic liver disease^31^ and identify high risk disease^32^. In addition, mpMRI has shown clinical utility in epidemiological studies of liver disease in asymptomatic adults and promoting earlier non-invasive disease detection^28,33,34^.

While pediatric clinical guidelines propose the need for accurate non-invasive MRI-based assessment of NAFLD, hitherto, there has been no data on the utility of mpMRI and the association of mpMRI metrics with metabolic and liver health assessments in young children, asymptomatic for NAFLD. Thus, in this study, we evaluated the relationship between mpMRI assessment of NAFLD/NASH with Body-Mass-Index (BMI), conventional biochemical liver function tests, and metabolic risk factors and score, in a pre-pubertal Mexican male cohort, asymptomatic for NAFLD. Male Hispanic populations have been shown to have both a higher prevalence of NAFLD/NASH as well as a greater likelihood of developing cirrhosis^35^ due to the high prevalence of the patatin-like phospholipase domain-containing protein 3 (PNPLA3)^36^. As males are more likely to be affected by NASH than females, the probability of poor prognosis is higher in males compared to females. Thus, in this study, we selected boys as their gender (sex) makes them highly susceptible to adverse outcomes from NASH as they grow older—these include 3.1 odds ratio of developing type 2 diabetes^37^, higher premature mortality rate^38^, and higher cumulative incidence of overall mortality^39^. Thus, as NASH is primarily a silent disease^40^, early detection can support initiation of non-therapeutic interventions, such as lifestyle changes, which can result in disease regression.We aim to determine the clinical usefulness of mpMRI for the assessment of NAFLD in asymptomatic boys who are at risk of having or developing advanced disease.

## Methods

### Study cohort

Male pre-pubertal individuals were invited to have a research non-contrast MRI scan as part of a study to identify predictors of the risk of developing metabolic and cognitive dysfunction (“METCOG” study). Participants were recruited from local schools, as well as from adverts posted in local newspapers and clinics at Hospital Infantil de México Federico Gómez, Mexico City, Mexico, and had differing BMI classifications based on percentiles as defined by the Centre of Disease Control and Prevention (CDC) guidelines for children and teenagers^41^. All participants were asymptomatic and did not have any reported/existing liver disease.

The study cohort comprised 81 participants who all underwent mpMRI. Statistical power analysis^42^ showed that a sample size of N = 78 is required for statistical significance. For this calculation, the diagnostic rate of mpMRI was considered to be comparable to that reported in adults^43^. Employing an effect size of 5% (determined by liver PDFF which was used to define NAFLD), with an acceptable level of significance of 5% (α = 0.05), 80% power and assuming a 5% dropout rate, a minimum of N = 78 participants were required to be recruited into the study.

The inclusion criteria were that individuals had to be male, aged 7–9 years old (Tanner stage 1), with the ability to hold their breath for 10–15 s whilst lying supine (for MRI). Exclusion criteria included having Type 1 diabetes, known or suspected liver disease, chronic disease except for obesity or metabolic syndrome, acute infection, taking metabolic profile-altering medication (e.g., metformin, corticosteroids, lipid lowering agents), first degree relative with Type 1 diabetes, hypoxia, psychiatric disorder or neurological disorders affecting normal cognitive development, as well as having metallic implants/devices that are MRI contraindicated or being claustrophobic.

**Fig. 1:**
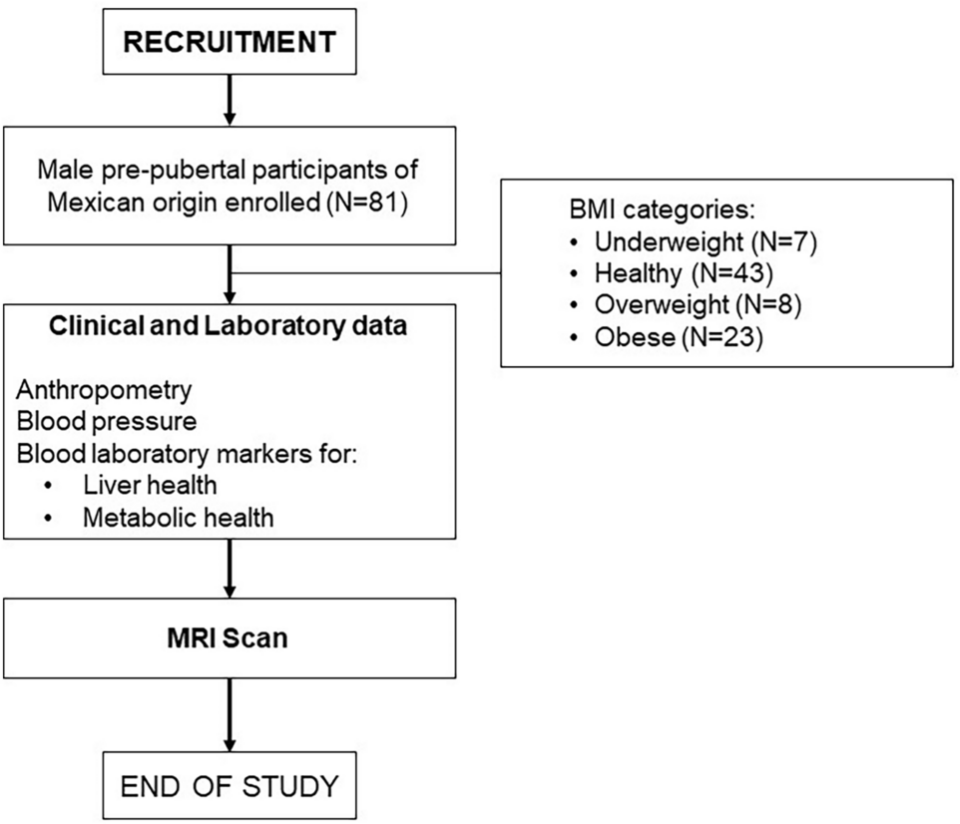
Study design for the “METCOG” study showing patient enrolment/exclusions and study visits and clinical, laboratory and imaging data collected.

Participants attended two visits as part of this study (Fig. 1): the first for anthropometric measurements and blood collection for laboratory tests; and the second for an MRI scan. Randomization was not undertaken, boys were enrolled as the study progressed and their measurements defined their BMI or liver health/disease group classification.

### Ethical approval

This cross-sectional observational study was carried out according to the Principals of Good Clinical Practice and the Declaration of Helsinki (2013). Ethical approval was received from the King’s College London Psychiatry, Nursing and Midwifery Research Ethics Subcommittee (ethics number: HR-16/17-4156) and from the Hospital Infantil de Mexico, Federico Gómez (ethics number: HIM/2016/105 and SSA-1369). All participants gave their written informed assent and their legal guardians, also gave written informed consent for the participants to take part in the study.

### Clinical and laboratory assessments

Anthropometric measurements including weight, height, waist circumference (WC) were recorded, alongside systolic and diastolic blood pressure (BP) readings. Fasted blood samples were collected, and plasma prepared for conventional liver biochemical measurements including alanine transaminase (ALT), aspartate transaminase (AST), alkaline phosphatase (ALP), total and direct bilirubin. Plasma markers of metabolic health including fasting insulin, lipid profile (high- and low-density lipoproteins [H/LDL], cholesterol, triglycerides) and hemoglobin A1c (HbA1c) were also measured. The Homeostatic Model Assessment for Insulin Resistance (HOMA-IR) was calculated according to the formula: fasting insulin (uU/L) × fasting glucose (nmol/L)/22.5.

BMI was calculated and individuals categorized using CDC classifications into low (underweight/healthy) and high (overweight/obese) BMI groups. Underweight was defined as a BMI ≤ 5th percentile, while healthy was defined as a BMI within the 5th to 85th percentiles. Overweight was defined as a BMI in the 85th to 95th percentiles, and obesity as a BMI ≥ 95th percentile. Participants were classified as prediabetic if their HbA1c measurement was between 5.7 and 6.4%, as advised by the American Diabetes Association^44^. Liver biopsy was not performed in this study as those recruited were not patients and therefore, not clinically indicated.

### Definition of metabolic syndrome (MetS) risk and NAFLD

Metabolic syndrome (MetS) is typically diagnosed in children above 10 years of age^45^. However, the ‘Identification and prevention of Dietary- and lifestyle-induced health Effects In Children and infantS’ (IDEFICS) study developed criteria which can be used to identify MetS in children younger than 10 years^38^. IDEFICS consider MetS risk factors in young children are: WC ≥ 90th percentile, high BP ≥ 90th percentile, dyslipidemia as triglycerides ≥ 90th percentile or HDL cholesterol ≤ 10th percentile and high blood glucose/insulin, with HOMA-IR ≥ 90th percentile or fasting glucose ≥ 90th percentile. In this study, the MetS risk for each participant was categorized into three groups according to the criteria developed in the IDEFICS study: no MetS risk factors, 1–2 MetS risk factors (some risk of MetS), and ≥ 3 MetS risk factors (indicative of likely MetS).

Participants were also defined and trichotomized into three groups, those with NAFLD, NASH or high-risk NASH, using mpMRI metric thresholds reported previously^26–30,32,46^. Those categorized as having NAFLD had PDFF of ≥ 5%; those as having NASH, PDFF was ≥ 5% and cT1 ≥ 800 ms; and those with high-risk NASH, i.e., NASH score of ≥ 4 and fibrosis stage (F) ≥ 2, PDFF was ≥ 5% and cT1 ≥ 875 ms^46^.

### MRI acquisition and analysis

**Fig. 2:**
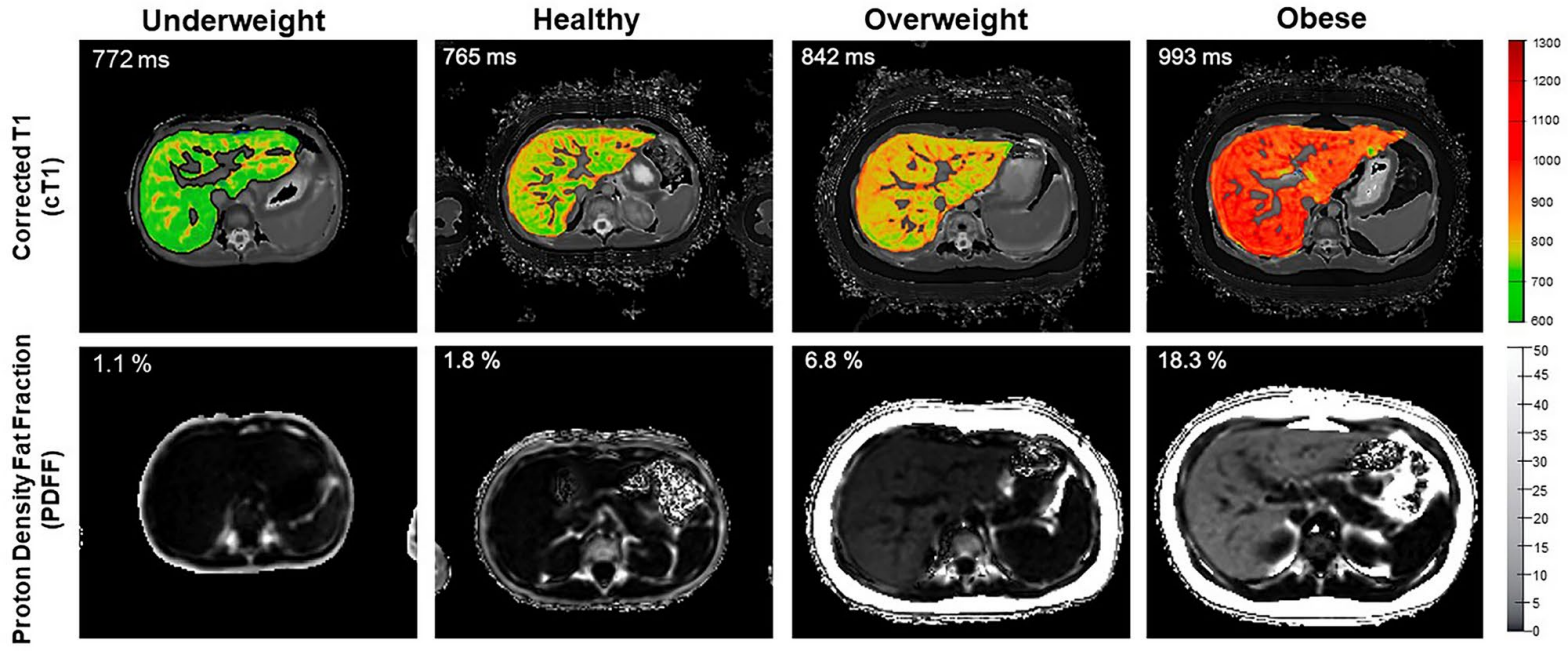
Typical transverse corrected T1 (cT1) and proton density fat fraction (PDFF) maps for underweight, healthy, overweight and obese participants calculated from multiparametric magnetic resonance imaging (mpMRI) data collected using LiverMultiScan (Perspectum Ltd, Oxford, UK) image acquisition protocol and MRI scanning sequences^44^.

MRI was performed on a 3 T Siemen’s Skyra scanner (Siemens Healthineers, Erlangen, Germany) with mpMR images obtained using a non-contrast abdominal MRI scan following the LiverMultiScan (Perspectum Ltd, Oxford, UK) image acquisition protocol with MRI scanning sequences reported previously^47^. Four transverse slices obtained at the porta hepatis location in the liver were acquired for each participant using a shortened modified look-locker inversion (shMOLLI) and a multi-echo spoiled gradient-echo sequence to quantify T1, iron (T2*) and fat (PDFF)^40^. During image analysis, iron-corrected T1 (cT1) and PDFF maps of the liver were delineated into whole liver segmentation maps (Fig. 2) using a semi-automatic method^47^. Three 15-mm diameter circular regions of interest were placed on the transverse T2* maps for each slice, covering a representative sample of the liver, to calculate average T2* values for T1-correction. All images were analyzed by trained analysts blinded to the clinical data.

### Statistical analysis

Descriptive statistics were used to summarize participant characteristics of the study cohort. Continuous normally distributed variables were reported as mean ± standard deviation (SD) and categorical variables as frequency and percentage, with ranges reported where appropriate. Correlations between measurements were investigated using Spearman’s Rank correlation coefficient (r_s_). Differences in measurement values between low and high BMI, or low and high cT1 groups, were compared using the Kruskal–Wallis rank sum test, and differences in categorical data were compared using either the Pearson’s Chi-squared or two-sided Fisher’s exact test as appropriate.

Statistical analysis was performed using R version 4.1.2 (R Core Team, Vienna, Austria) with values of p < 0.05 considered statistically significant. For the mpMRI and MetS risk sub-analyses, where data were missing, case-wise deletion was employed.

## Results

### Participant demographics

The study cohort (n = 81) were aged 8.2 ± 0.8 years (range 7.0–9.9) with mean BMI of 17.4 ± 3.5 kg/m^2^ (range 12.9–29.4). Clinical assessment included collection of anthropometric, BP, and plasma metabolic and liver measurements, alongside a non-contrast MRI scan (Table 1). All participants were asymptomatic and did not have any reported/existing liver disease.

**Table 1:** Age, anthropometric, blood pressure and laboratory measurements from the study cohort, and low and high Body-Mass-Index (BMI) subgroups.

Measurement	Whole cohort n = 81	Low BMI Subgroup n = 50	High BMI Subgroup n = 31	Low vs. high BMI P-value
Age (years)	8.2 (0.8)	8.2 (0.8)	8.2 (0.7)	0.7
Anthropometric measurements				
BMI (kg/m^2^)	17.4 (3.5)	15.3 (1.2)	20.9 (3.1)	** < 0.001**
Waist circumference (cm)	63.3 (10.0)	57.1 (4.0)	73.2 (8.8)	** < 0.001**
Waist/height ratio	0.5 (0.1)	0.5 (0.0)	0.6 (0.1)	** < 0.001**
Metabolic health measurements				
Systolic blood pressure (mmHg)	99.0 (6.4)	97.2 (5.7)	102.0 (6.5)	** < 0.001**
Diastolic blood pressure (mmHg)	61.0 (5.6)	59.4 (4.9)	63.7 (5.7)	**0.001**
Glucose (mg/dL)	85.7 (7.4)	85.1 (7.6)	86.6 (7.0)	0.24
Fasting insulin (pg/mL)	213.8 (209.6)	165.8 (87.5)	287.4 (304.5)	**0.025**
HOMA-IR score	1.3 (1.5)	1.0 (0.5)	1.9 (2.2)	**0.021**
HbA1c (%)	5.3 (0.3)	5.3 (0.3)	5.3 (0.3)	0.38
Prediabetics (HbA1c 5.7–6.4%)	9 (11%)	3 (6.0%)	6 (19%)	0.079
Leptin (pg/mL)	2,446.8 (2,940.8)	1,033.3 (964.8)	4,617.5 (3,592.4)	** < 0.001**
HDL (mg/dL)	57 (26)	63 (31)	46 (12)	** < 0.001**
LDL (mg/dL)	94 (23)	88 (18)	103 (27)	**0.008**
Triglycerides (mg/dL)	78.7 (74.1)	57.1 (22.7)	113.5 (108.5)	**0.003**
Plasma liver biochemical tests				
AST (U/L)	29 (6)	28 (6)	32 (6)	**0.002**
ALT (U/L)	32 (15)	28 (9)	38 (21)	**0.008**
ALP (U/L)	288.6 (63.2)	270.7 (58.1)	316.8 (61.3)	**0.001**
Direct bilirubin (mg/dL)	0.10 (0.03)	0.10 (0.03)	0.09 (0.02)	**0.041**
Total bilirubin (mg/dL)	0.48 (0.16)	0.51 (0.19)	0.44 (0.09)	0.15

Significant values are in bold.

Differences between low and high BMI groups considered significant are denoted in bold. BMI and prediabetes classification were performed according to the Centre for Disease Control classification and American Diabetes Association, respectively. *HDL* high-density lipoprotein, *LDL* low-density lipoprotein, *AST* aspartate aminotransferase, *ALT* alanine aminotransaminase, *ALP* alkaline phosphatase, *HOMA-IR* Homeostatic Model Assessment for Insulin Resistance.

### Metabolic and liver health

**Fig. 3:**
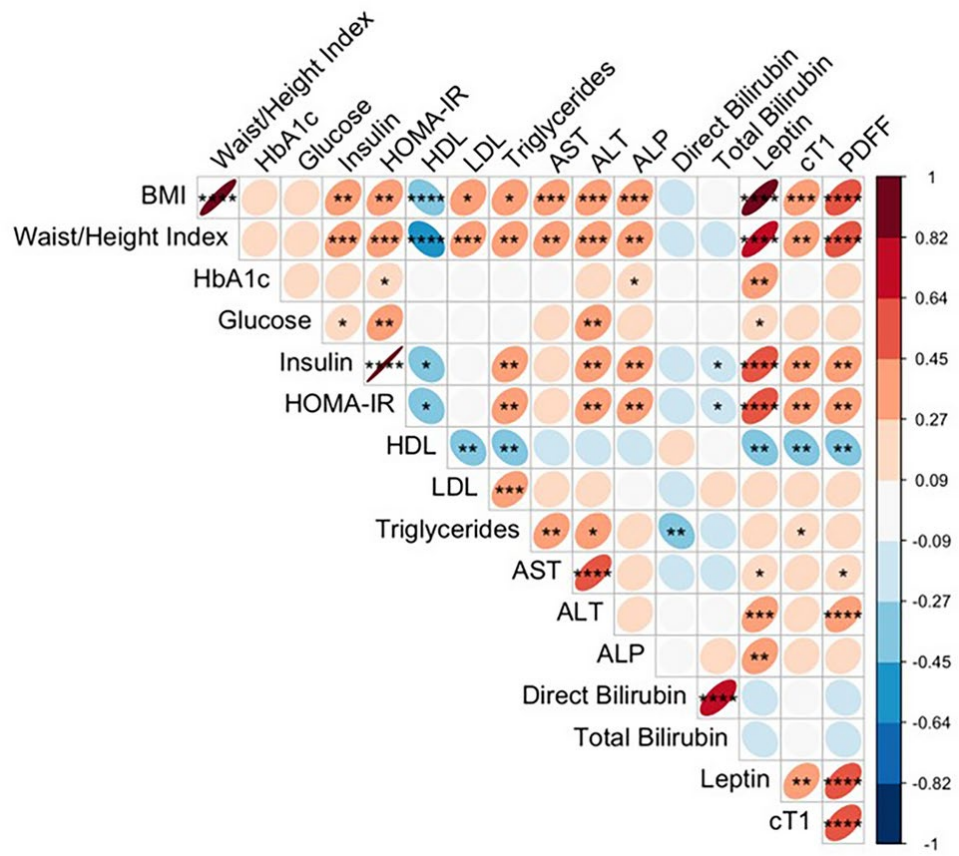
A correlation plot showing the associations between anthropometric, plasma liver and metabolic, and multiparametric magnetic resonance imaging measurements. Ellipse area reflect the absolute value of the corresponding Spearman’s correlation coefficient, and their eccentricity is parametrically scaled to the correlation value. All significant correlations are denoted in bold with the following levels of significance: *p < 0.05; **p < 0.01; ***p < 0.001; ****p < 0.0001. *BMI* Body-Mass-Index, *HbA1c* hemoglobin A1c, *HOMA-IR* Homeostatic Model Assessment for Insulin Resistance, *HDL* high-density lipoprotein, *LDL* low-density lipoprotein, *AST* aspartate aminotransferase, *ALT* alanine aminotransaminase, *ALP* alkaline phosphatase, *cT1* corrected T1, *PDFF* proton density fat fraction.

BMI, cT1 and PDFF had significant correlations with anthropometric parameters, measures of metabolic and liver health (Fig. 3). BMI correlated with all measures except for HbA1c, glucose, direct and total bilirubin. Whilst both cT1 and PDFF correlated with BMI, and with each other, they also correlated with waist/height index, insulin, HOMA-IR, HDL and leptin. Additionally, PDFF (liver fat) also correlated with AST and ALT, and cT1 with triglycerides.

**Fig. 4:**
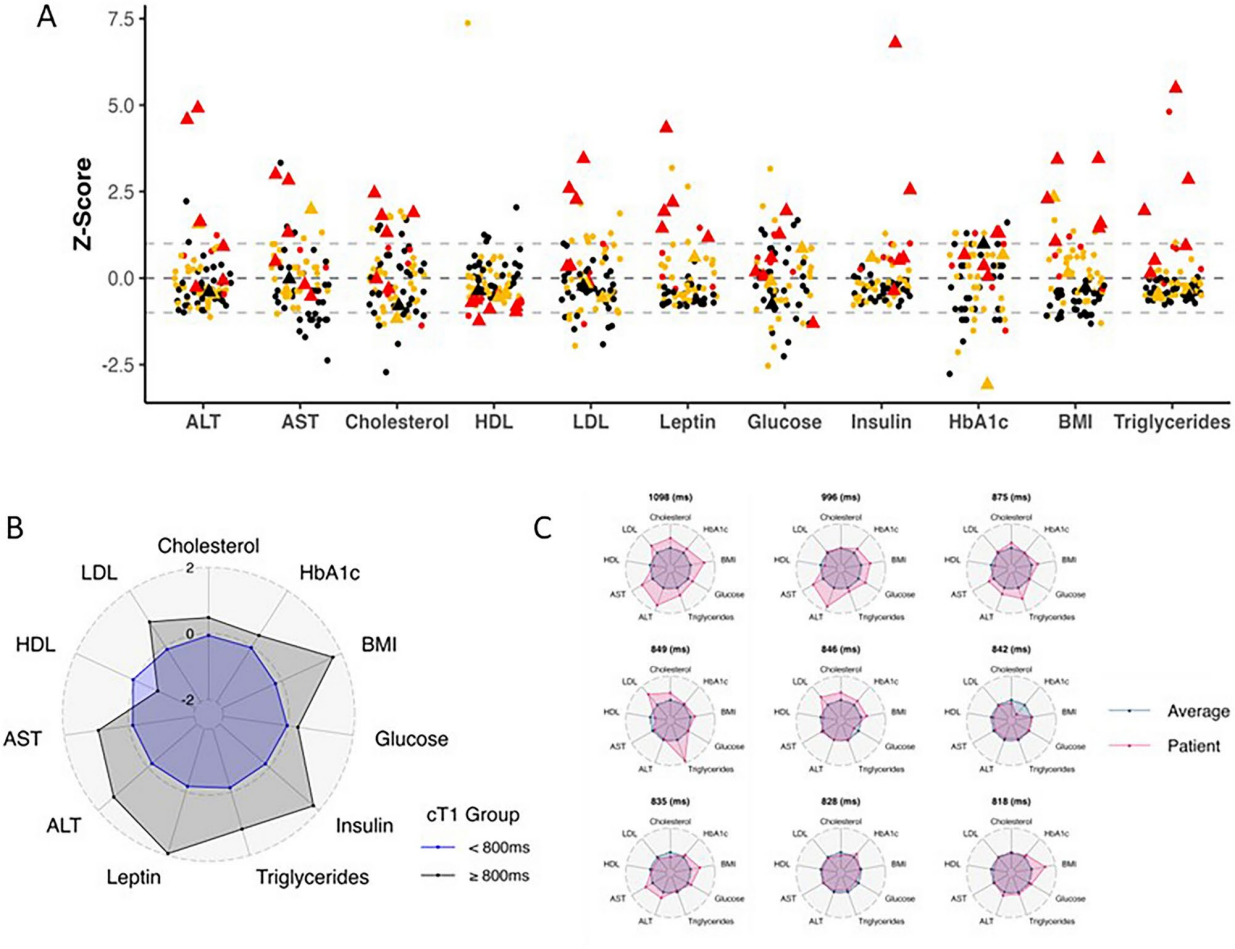
Radar plots showing multivariate observations for group-wise comparison between those with low (< 800 ms) and elevated cT1 (≥ 800 ms) (**A**), and with individual children with elevated cT1 (**B**). (**C**) A Manhattan plot showing individual z-scores for selected markers of metabolic and liver health (dashed lines represent the mean and standard deviation of the z-scores), and their relationship to the metabolic syndrome (MetS) risk score (Key: black, orange and red symbols indicate no MetS; some risk of MetS, 1–2 risk factors; and likely MetS, ≥ 3 risk factors, respectively); and a circle or a triangle indicate cT1 < 800 ms or ≥ 800 ms, respectively. *BMI* Body-Mass-Index, *HbA1c* hemoglobin A1c, *HOMA-IR* Homeostatic Model Assessment for Insulin Resistance, *HDL* high-density lipoprotein, *LDL* low-density lipoprotein, *AST* aspartate aminotransferase, *ALT* alanine aminotransaminase, *ALP* alkaline phosphatase, *cT1* corrected T1, *PDFF* proton density fat fraction.

Of the study cohort, 38% had high BMI (overweight/obese). Whilst most plasma biochemical markers were in reported normal ranges^48^, 11% had HbA1c in the prediabetic range (Table 2). The high BMI group had higher fasting insulin (p = 0.001) and triglyceride levels (p < 0.001), alongside lower serum HDL (p < 0.001), than the low BMI group (Table 1). Similarly, biochemical markers of liver health were also in the normal range, but AST (p = 0.023), ALT (p < 0.001), ALP (p = 0.008) and direct bilirubin (p = 0.015), were higher in the high BMI group compared to the low BMI group. In addition, both diastolic (p = 0.006) and systolic (p = 0.013) BP were also higher in the high BMI group (Table 1). Inspection of a Manhattan plot where individual datapoints are shown for the liver transaminases and selected measurements that contribute to the IDEFCS MetS risk score, many of the abnormal values are from subjects with MetS risk score of 1–2 or ≥ 3, but some are also from subjects that have no MetS risk factors (Fig. 4A).

**Table 2:** Anthropometric and liver measurements, and their comparisons between low and high Body-Mass-Index (BMI), and between low and high cT1, subgroups.

Variable	Whole cohort n = 81	Low BMI subgroup n = 50	High BMI subgroup n = 31	Low vs High BMI P-value	cT1 < 800 ms subgroup n = 72	cT1 $\ge$ 800 ms subgroup n = 9	Low vs. high cT1 P-value
Age (years)	8.2 (0.8)	8.2 (0.8)	8.2 (0.7)	0.7	8.2 (0.8)	8.6 (0.7)	0.12
Anthropometric measurements							
BMI (kg/m^2^)	17.4 (3.5)	15.3 (1.2)	20.9 (3.1)	**< 0.001**	16.7 (2.5)	23.4 (4.5)	**< 0.001**
High BMI (%)	31 (38%)	0 (0%)	31 (100%)	**< 0.001**	23 (32%)	8 (89%)	**0.002**
Waist/height ratio	0.5 (0.1)	0.5 (0.0)	0.6 (0.1)	**< 0.001**	0.5 (0.1)	0.6 (0.1)	**< 0.001**
Plasma liver biochemical tests							
AST (U/L)	29 (6)	28 (6)	32 (6)	**0.002**	28 (5)	35 (8)	**0.033**
ALT (U/L)	32 (15)	28 (9)	38 (21)	**0.008**	29 (9)	53 (32)	**0.01**
ALP (U/L)	288.6 (63.2)	270.7 (58.1)	316.8 (61.3)	**0.001**	284.9 (64.5)	316.7 (45.7)	0.076
Direct bilirubin (mg/dL)	0.10 (0.03)	0.10 (0.03)	0.09 (0.02)	**0.041**	0.10 (0.03)	0.09 (0.02)	0.39
Total bilirubin (mg/dL)	0.48 (0.16)	0.51 (0.19)	0.44 (0.09)	0.15	0.49 (0.16)	0.41 (0.09)	0.11
Multiparametric magnetic resonance imaging metrics and liver health/disease assessment							
cT1 (ms)	758 (62)	738 (31)	790 (83)	**< 0.001**	742 (30)	887 (95)	**< 0.001**
PDFF (%)	3.5 (3.5)	2.2 (0.8)	5.5 (4.9)	**< 0.001**	2.6 (1.3)	10.0 (7.2)	**< 0.001**
NAFLD (PDFF ≥ 5%)	10 (12%)	0 (0%)	10 (32%)	**< 0.001**	4 (5.6%)	6 (67%)	**< 0.001**
NASH (PDFF ≥ 5% and cT1 ≥ 800 ms)	6 (7.4%)	0 (0%)	6 (19%)	**0.002**	0 (0%)	6 (67%)	**< 0.001**
High risk NASH (PDFF ≥ 5% and cT1 ≥ 875 ms)	3 (3.7%)	0 (0%)	3 (9.7%)	0.053	0 (0%)	3 (33%)	**< 0.001**

Significant values are in bold.

BMI category was determined according to the Centre for Disease Control classification. *AST* aspartate aminotransferase, *ALT* alanine aminotransaminase, *ALP* alkaline phosphatase, *cT1* corrected T1, *PDFF* proton density fat fraction.

### Liver mpMRI, BMI, metabolic syndrome factors and risk

**Fig. 5:**
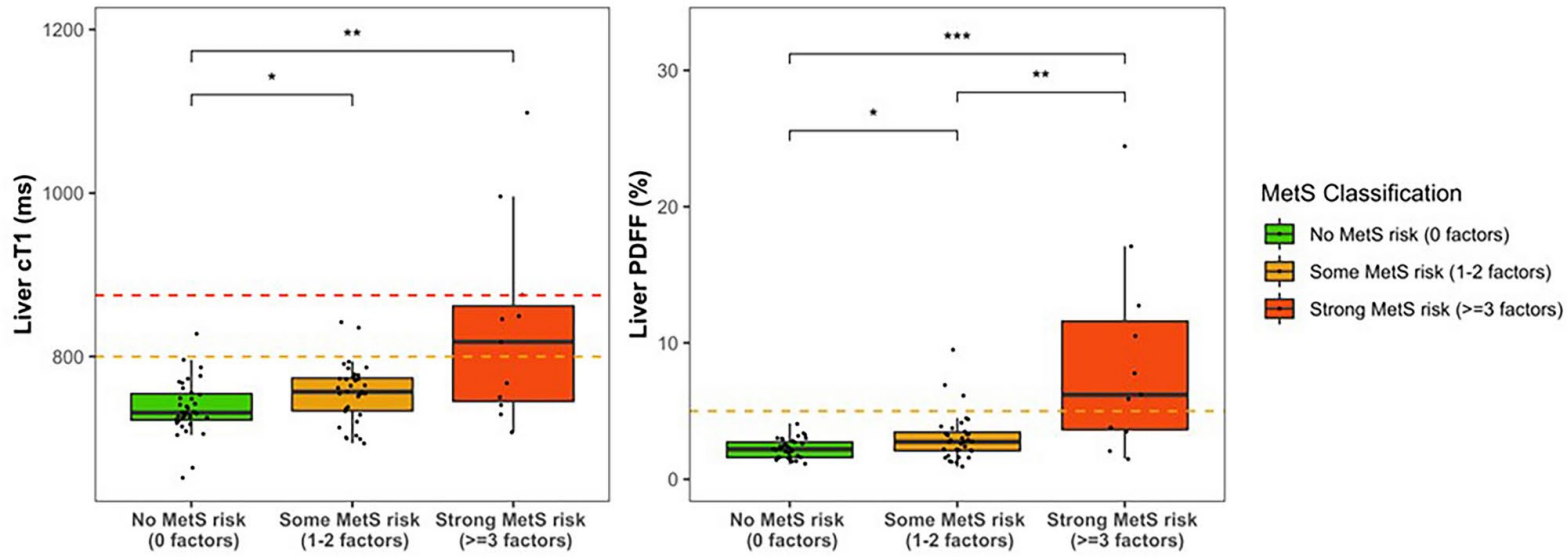
The relationship between corrected T1 (cT1), proton density fat fraction (PDFF) and increasing metabolic syndrome (MetS) risk groups. (**A**) Graphs showing the cT1 in groups with different numbers of metabolic syndrome (MetS) risk factors; and (**B**), A radar plot illustrating the levels of liver (alanine and aspartate) transaminases and selected metabolic parameters contributing to MetS risk factor scores with cT1 < 800 ms and those with cT1 ≥ 800 ms, irrespective of MetS risk factor score. All significant Spearman’s correlations are denoted in bold with the following levels of significance: *p < 0.05; **p < 0.01; ***p < 0.001.

In this cohort, employing thresholds defined previously in literature^26–30,32,39^, 12% of the cohort had NAFLD (PDFF ≥ 5%), 7% had NASH (both PDFF ≥ 5% and cT1 ≥ 800 ms) and 4% high-risk NASH (both PDFF ≥ 5% and cT1 ≥ 875 ms) (Table 2). Both PDFF and cT1 were higher in the high BMI group (p < 0.001) (Table 2). When considering the individual components of MetS risk, individuals with elevated cT1 (≥ 800 ms) had significantly greater hyperinsulinemia/glycaemia, hyperlipidemia and low HDL when compared to those with cT1 < 800 ms (Table 3). In addition, children with higher cT1 also had significantly elevated fasting insulin, triglycerides and HOMA-IR (Table 3). Fasting leptin was also significantly higher in those with high BMI as well as those with elevated cT1 (Table 3). Figure 5 shows the relationship between cT1 and PDFF with MetS risk. Participants with ≥ 3 MetS risk factors (likely MetS) had significantly higher cT1 compared to those without any MetS risk factors (834 ± 121 ms vs 737 ± 33 ms, p = 0.047) (Table 3). Indeed, the odds ratio of having cT1 ≥ 800 ms were 7.4, 12.8, 24.5 and 49.8 for MetS risk scores, 0, 1, 2, and ≥ 3, respectively. This is also consistent with the radar plot, where those that have cT1 < 800 ms shows relatively less perturbation of plasma liver and selected metabolic measures compared to those that have high cT1 on average (Fig. 4B), and individually (Fig. 4C). Inspection of the Manhattan plot of individual measurement values for each subject individual, show that not all individuals with high cT1 had high BMI, increased MetS risk, abnormal liver or metabolic measures (Fig. 4A).

**Table 3:** Metabolic health measurements, and Identification and prevention of dietary- and lifestyle-induced health effects in children and infants (IDEFICS) metabolic syndrome (MetS) risk factors categories and MetS risk criteria; and their comparisons between low and high Body-Mass-Index (BMI), and between low and high cT1, subgroups.

Variable	Whole cohort n = 81	Low BMI subgroup n = 50	High BMI subgroup n = 31	Low vs High BMI P-value	cT1 < 800 ms subgroup n = 72	cT1 $\ge$ 800 ms subgroup n = 9	Low vs. High cT1 P-value
Metabolic health measurements							
Systolic blood pressure (mmHg)	99.0 (6.4)	97.2 (5.7)	102.0 (6.5)	** < 0.001**	98.7 (6.3)	101.7 (6.8)	0.18
Diastolic blood pressure (mmHg)	61.0 (5.6)	59.4 (4.9)	63.7 (5.7)	**0.001**	60.6 (5.4)	64.6 (6.1)	0.078
Fasting glucose (mg/dL)	85.7 (7.4)	85.1 (7.6)	86.6 (7.0)	0.24	85.4 (7.4)	87.9 (7.4)	0.35
Fasting insulin (pg/mL)	213.8 (209.6)	165.8 (87.5)	287.4 (304.5)	**0.025**	179.3 (99.7)	586.6 (552.9)	**0.005**
HOMA-IR score	1.3 (1.5)	1.0 (0.5)	1.9 (2.2)	**0.021**	1.1 (0.6)	4.0 (4.1)	**0.005**
HbA1c (%)	5.3 (0.3)	5.3 (0.3)	5.3 (0.3)	0.38	5.3 (0.3)	5.4 (0.4)	0.054
Prediabetics	9 (11%)	3 (6.0%)	6 (19%)	0.079	6 (8.3%)	3 (33%)	0.058
Leptin (pg/mL)	2,446.8 (2,940.8)	1,033.3 (964.8)	4,617.5 (3,592.4)	** < 0.001**	1,918.9 (2,228.6)	8,165.7 (3,825.4)	** < 0.001**
Total cholesterol (mg/dL)	162.7 (24.5)	159.5 (24.6)	167.9 (23.9)	0.15	161.2 (23.0)	174.6 (33.9)	0.38
Triglycerides (mg/dL)	78.7 (74.1)	57.1 (22.7)	113.5 (108.5)	**0.003**	67.8 (51.8)	165.6 (146.8)	**0.022**
HDL (mg/dL)	57 (26)	63 (31)	46 (12)	** < 0.001**	59 (27)	37 (7)	** < 0.001**
LDL (mg/dL)	94 (23)	88 (18)	103 (27)	**0.008**	91 (20)	114 (34)	0.055
IDEFICS MetS risk factors and mets risk assessment							
Obesity: ≥ 90th percentile as assessed by WC	37 (46%)	6 (12%)	31 (100%)	** < 0.001**	29 (40%)	8 (89%)	**0.01**
Blood pressure: systolic or diastolic ≥ 90th percentile	4 (4.9%)	0 (0%)	4 (13%)	**0.019**	2 (2.8%)	2 (22%)	0.059
Glucose: Insulin or fasting glucose ≥ 90th percentile	17 (21%)	7 (14%)	10 (32%)	**0.05**	12 (17%)	5 (56%)	**0.017**
Triglycerides: ≥ 90th percentile	17 (21%)	5 (10%)	12 (39%)	**0.002**	11 (15%)	6 (67%)	**0.002**
HDL Cholesterol: ≤ 10th percentile	9 (11%)	0 (0%)	9 (29%)	** < 0.001**	4 (5.6%)	5 (56%)	** < 0.001**
No MetS risk	36 (44%)	36 (72%)	0 (0%)	** < 0.001**	35 (49%)	1 (11%)	**0.039**
Some MetS risk	34 (42%)	13 (26%)	21 (68%)	** < 0.001**	32 (44%)	2 (22%)	0.29
MetS $\ge$ 3 risk factors	11 (14%)	1 (2.0%)	10 (32%)	** < 0.001**	5 (6.9%)	6 (67%)	** < 0.001**

Significant values are in bold.

BMI and prediabetes classification were performed according to the Centre for Disease Control classification and American Diabetes Association, respectively. *HbA1c* hemoglobin A1c, *HOMA-IR* Homeostatic Model Assessment for Insulin Resistance, *HDL* high-density lipoprotein, *LDL* low-density lipoprotein.

Similar to that for cT1, PDFF (liver fat) was found to increase with increasing risk of MetS such that individuals with ≥ 3 MetS risk factors had significantly higher PDFF compared to those without any risk factors (8.7 ± 7.1% vs 2.2 ± 0.7%, p < 0.001). In addition to having elevated liver health markers (biochemical and imaging), participants with high BMI had significantly higher diastolic and systolic (p = 0.001) BP compared to their counterparts with low BMI (Tables 1, 3).

## Discussion

In this study, we showed high BMI, increased MetS factors and risk scores, and liver transaminases, with elevated mpMRI metrics, in a Hispanic pre-pubertal children cohort. Notably, liver transaminases were within normal ranges and consistent with their insensitivity to early NAFLD. To the best of our knowledge, this is the first study investigating the relationship between BMI (obesity), biochemical measurements of liver health, metabolic disease risk and mpMR metrics in a young asymptomatic Hispanic pediatric population, predisposed to developing NAFLD.

The pathogenesis of NAFLD and progression to NASH is associated with the dysregulation of metabolic parameters^3^. Adiposity, insulin resistance and hyperlipidemia, all characteristics of MetS have been shown to increase individual risk of NAFLD^1,2^. BMI has traditionally been used as part of the criteria to identify children with highest risk of long-term adverse outcomes, thus, guiding the intensity of intervention^9,10^. Findings from this study corroborated with these insights as children with high BMI (overweight/obese) and elevated metabolic risk markers showed increased PDFF and cT1; and notably, with plasma liver health measurements generally in the normal range. However, although BMI is closely associated with NAFLD, those with low BMI can still develop the disease, and as NAFLD is typically asymptomatic in its early stages, with liver health measurements such as ALT and AST being within the normal range^10^, mpMRI may play a critical role in identifying such individuals.

Large patient registries have recently shown that patients with NASH, regardless of their fibrosis stage, are at high risk of poor clinical outcomes^48,49^. Furthermore, the burden of NASH on healthcare systems and the economy has been acknowledged to be significant on a global scale^50,51^, and that early diagnosis and treatment of NASH patients could potentially reduce future healthcare costs. Therefore, early identification of individuals with NASH, with or without fibrosis, is important to provide more effective prevention, surveillance, and intervention strategies. Non-invasive technologies are currently being used to support adult management and triaging in NAFLD/NASH^52,53^, and to enrich and assess treatment response in adult pharmaceutical clinical trials^27^. This is also needed in the pediatric arena as systematic reviews on the global prevalence of NAFLD have indicated rates of 7.6% in the general pediatric population and 34.2% in the obese population^54^. Furthermore, epidemiological studies have shown NASH prevalence range from 20 to 50% in these NAFLD populations^48^.

In adults with NAFLD, mpMRI markers (cT1 and PDFF) have been reported to show good diagnostic utility to stratify NASH with fibrosis (with an AUC ranging 0.74–0.89)^32,46,55,56^; outperform other commonly used non-invasive markers, including biochemical markers, vibration-controlled transient elastography and magnetic resonance elastography^32^; perform similarly in different ethnic and racial cohorts (including black, white, Asian and Hispanic)^32,46,55,56^; and have the same performance as liver biopsy in predicting adverse clinical outcomes^26^. In our study, 12% had elevated cT1 indicative of NAFLD, with 7% suggested to have NASH, and 4%, high-risk NASH. High-risk NASH, i.e., NASH with fibrosis, has been linked to severe adverse clinical events including poor cardiovascular outcomes^57^. In this asymptomatic cohort, children with high cT1 also had significantly higher (but within the normal range) transaminase levels as well as increased MetS risk compared to those with low cT1 (< 800 ms). As these MRI markers seem to perform similarly in children as in adults, we suggest that cT1 can be used for early disease diagnosis and stratify children for appropriate intervention. Interventions include more robust weight-loss intervention, or treatment with drug therapies licensed for diabetes or weight-loss that have beneficial effects in the liver as proposed in adults^52,58^.

Elevated cT1 and PDFF are especially important clinical findings in this cohort as male Hispanic populations have higher prevalence of NAFLD/NASH and greater risk of developing cirrhosis^35^. These findings are further strengthened by the significant associations between cT1 and PDFF with leptin, an independent predictor of the presence and development of NAFLD, and strongly associated with insulin resistance and body adiposity^59^. It is worth noting that both pediatric and adult clinical guidelines are demanding studies that evaluate the clinical utility of non-invasive technologies that can be used to identify early NAFLD/NASH. Regulatory bodies, such as the FDA, are also calling for non-invasive technologies as only 3% of FDA-approved artificial imaging solutions are indicated in pediatrics^60^. Our study demonstrates the potential added benefit of mpMRI by characterizing the relationship between metabolic and liver health, suggesting mpMRI can be used to provide early liver disease detection and support clinical management. This is especially relevant as recent literature authored by members of the European Reference Network for Hepatological Diseases have described mpMRI as a “virtual biopsy” that not only provides a panoramic view of the liver but also facilitates risk stratification of patients^61^.

The term “non-alcoholic” to describe metabolic-associated liver disease related to overweight/obesity and metabolic syndrome in children has been the subject of much debate, especially as the possibility of alcohol-related liver disease is unlikely^62^. In recognition of this, some experts have proposed the use of the term “metabolic (dysfunction)-associated fatty liver disease” (MAFLD). However, the characterization of MAFLD in pediatrics is still poorly understood^62^, and the manifestation of MAFLD in children differs from that in adults^62^, increasing the complexity of disease screening. Nevertheless, while avoiding this ongoing controversy, our findings presented here contribute to these current discussions and support the development of clinical algorithms combining traditional biomarkers with mpMRI to improve diagnostic accuracy and obviate the need for invasive diagnostic liver biopsies^62^. These algorithms for non-invasive early detection of disease, have the potential to reduce the health consequences in the long-term associated with chronic childhood liver disease^63^.

This study has several strengths. Our findings highlight the potential relationships between mpMRI-assessed NAFLD/NASH with BMI, metabolic health and conventional liver assessments in an unusual cohort. Not only are findings from pre-pubertal children scarce, findings from a cohort living in a low- to middle-income countries such as Mexico is also uncommon. Furthermore, this study is important as amongst Hispanic persons, NAFLD is the most prevalent chronic liver disease, resulting in high incidences of hepatocellular carcinoma with worse prognosis compared to other ethnicities^64^.

The study does have a limitation, in that as it was a real-world study, concurrent liver biopsy was unethical in asymptomatic children. We acknowledge we are unable to assess investigated relationships with histological findings of NAFLD/NASH. Nevertheless, previous studies have shown significant correlations between cT1 and histology in both pediatrics^19–21^ and adults^26,27,32^.

In conclusion, the increasing prevalence of pediatric obesity has been strongly linked with rising global rates of NAFLD. This prospective study has demonstrated that elevated cT1 and PDFF, indicating liver dysfunction, are correlated with higher BMI and a greater risk of MetS. We propose non-invasive cT1 has clinical utility in routine pediatric NAFLD screening programs for NAFLD and its severity, alongside fibrosis markers for those with advanced disease. This would support stratification of young asymptomatic children at increased risk of developing NAFLD, e.g. those with high BMI and increased metabolic disease risk, and has the potential to enable early disease detection and reduce adverse clinical outcomes in young children.

## Data Availability

The data and analytic methods used in this study remain the property of the individual study sponsors. All deidentified participant data are not openly available presently to allow privileged use of the data to the funded researchers and may be made available to other researchers upon request to P-WS, following permission, investigator support and a signed data access agreement. Study metadata will be made available through the MRC Research Data Gateway to allow potential users to make contact and request access to the data.
